# Unexpected one-pot formation of the 1*H*-6a,8a-epiminotricyclopenta[*a*,*c*,*e*][8]annulene system from cyclopentanone, ammonia and dimethyl fumarate. Synthesis of highly strained polycyclic nitroxide and EPR study

**DOI:** 10.3762/bjoc.15.259

**Published:** 2019-11-07

**Authors:** Sergey A Dobrynin, Igor A Kirilyuk, Yuri V Gatilov, Andrey A Kuzhelev, Olesya A Krumkacheva, Matvey V Fedin, Michael K Bowman, Elena G Bagryanskaya

**Affiliations:** 1 N. N. Vorozhtsov Novosibirsk Institute of Organic Chemistry SB RAS, Lavrentiev Ave. 9, Novosibirsk, 630090, Russia; 2 Novosibirsk State University, Pirogova Str. 2, Novosibirsk, 630090, Russia; 3 International Tomography Center SB RAS, Institutskaya Str. 3a, Novosibirsk, 630090, Russia; 4 University of Alabama, Tuscaloosa, Alabama 35487-0336, United States

**Keywords:** domino reactions, EPR, nitroxide, spin relaxation

## Abstract

The unexpected formation of a highly strained polycyclic amine was observed in a one-pot synthesis from cyclopentanone, dimethyl fumarate and ammonium acetate. This multistep reaction includes 1,3-dipolar cycloaddition of dimethyl fumarate to the cyclic azomethine ylide formed in situ from cyclopentanone and ammonia. The polycyclic amine product was easily converted into a sterically shielded polycyclic nitroxide. The EPR spectra and spin relaxation behavior of the nitroxide were studied in solution. The spin relaxation seems well suited for the use as a biological spin label and are comparable with those of cyclic nitroxides with two spirocyclic moieties adjacent to the N–O**^·^** group.

## Introduction

Domino reactions have attracted much attention as an approach for the synthesis of complex molecules in a few steps [1]. The utility of multicomponent reactions involving amines, activated olefins and carbonyl compounds for the synthesis of heterocyclic compounds has been repeatedly demonstrated [2–3]. We recently used a domino reaction of amino acid, ketone and dimethyl fumarate for the one-pot synthesis of a substituted pyrrolidine, which then was converted into a reduction-resistant pyrrolidine nitroxide [4]. Here we report the unexpected formation of a highly strained polycyclic amine from cyclopentanone, dimethyl fumarate and ammonium acetate. This multistep reaction obviously includes the 1,3-dipolar cycloaddition of dimethyl fumarate with cyclic azomethine ylide formed in situ from cyclopentanone and ammonia. The polycyclic amine product was then converted into a sterically shielded polycyclic nitroxide.

Sterically hindered nitroxides have high chemical stability [5] and can be used as spin labels to study biopolymers in cells [6]. The introduction of spirocyclic moieties has a smaller effect on the reduction rates of nitroxides than does the introduction of linear alkyl substituents. However, spirocyclic nitroxides may have much longer spin relaxation times at 70–150 K which make them attractive agents for spin labeling [7–9]. Sterically hindered nitroxides can be used as spin labels for measurements at room temperature [10]. In this paper we examined the properties of this new nitroxide, in particular, the electron spin relaxation at different temperatures in water/glycerol solution.

## Results and Discussion

### Synthesis

#### Polycyclic amine

**Scheme 1:**
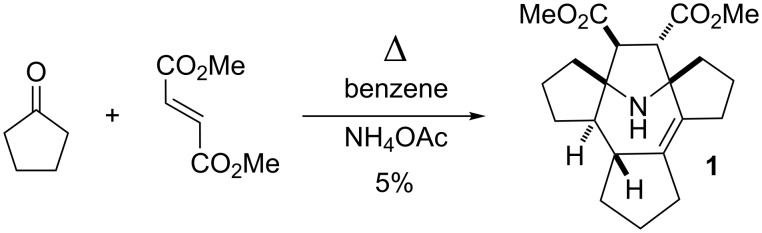
Synthesis of compound **1**.

**Figure 1:**
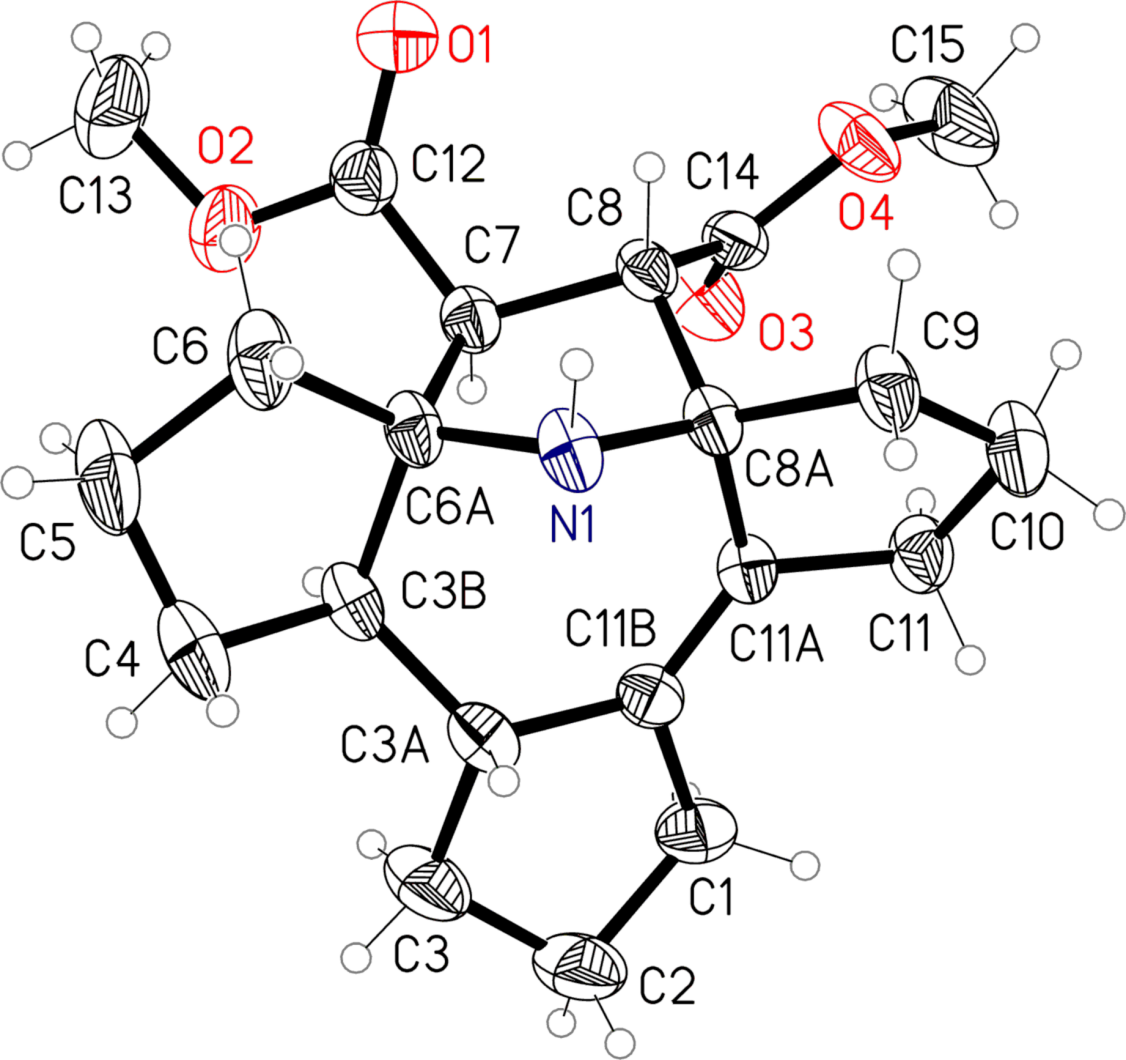
X-ray structure of compound **1** (one of the two enantiomers present in the crystal).

**Scheme 2:**
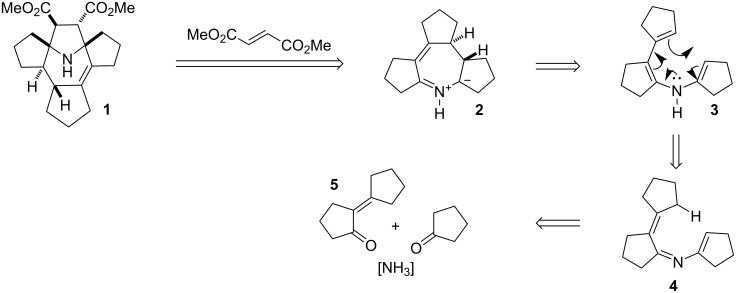
Possible mechanism for the formation of **1**.

A mixture cyclopentanone, dimethyl fumarate and ammonium acetate was refluxed in benzene in a Dean–Stark apparatus. The reaction mixture underwent a strong tarring and after standard extraction with aqueous sulfuric acid, the main product **1** was isolated in a low yield (ca. 5%, see Scheme 1). In another experiment a much higher yield was obtained (ca. 15%), however, we failed to reproduce this result and in subsequent experiments the yields were close to the 5% limit. The ^1^H NMR spectra of **1** show signals of two ester groups and multiple methylene and methyne protons with an overall intensity of 22 protons. The ^13^C NMR spectrum showed signals from 21 carbon atoms, including an isolated, fully substituted C=C moiety at 133.5 and 137.5 ppm, methoxy groups at 51.5 and 51.6 ppm and carboxylate groups at 172.7 and 175.3 ppm, respectively. There are also four signals originating from CH groups at 45.9, 55.1, 59.9 and 60.4 ppm. The two downfield CH groups belong to isolated spin systems in the ^1^H NMR spectra at 3.19 and 3.44 ppm with hfs constants of 3 Hz. In the HMBC spectrum, the latter protons show cross peaks with the carboxylate carbons and with nodal carbons at 76.08 and 76.12 ppm. The above data imply the presence of a 2,2,5,5-tetrasubstituted-3,4-bis(methoxycarbonyl)pyrrolidine ring. The ^1^H signal at 3.19 ppm shows a cross peak with an atom of a fully substituted C=C moiety, while another lower field signal of a CH group at 3.44 ppm interacts with a CH-group carbon at 55.1 ppm. The hydrogen of the latter group shows cross peaks with the carbon of the ^3a^CH group at 45.9 ppm and with the carbon of the ^11b^C=C moiety at 137.5 ppm. These interactions clearly indicate a 9-azabicyclo[4.2.1]non-2-ene system in the structure of **1**. The remaining assignments were made on the basis of HSQC, COSY ^1^H,^1^H and ^1^H,^13^C NMR spectra (see Table 1). The structure of the compound was finally confirmed by X-ray analysis and elemental analysis data (Figure 1). A possible mechanism for the formation of **1** is presented in Scheme 2.

**Table 1:** NMR Assignment of **1** based on the HSQC, COSY ^1^H,^1^H and ^1^H,^13^C spectra.

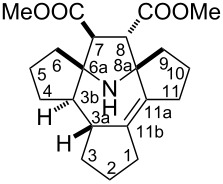					

	^13^C (ppm)	^1^H (ppm)		^13^C (ppm)	^1^H (ppm)

^10^CH_2_	21.0	1.71; 2.00	^3a^CH	45.9	2.45
^2^CH_2_	24.1	1.43; 1.68	CH_3_	51.5; 51.6	3.57; 3.66
^5^CH_2_	25.3	1.51; 1.63	^3b^CH	55.1	2.39
^11^CH_2_	30.1	1.93; 2.24	^7^CH	59.9	3.44
^1^CH_2_	32.4	2.01; 2.15	^8^CH	60.4	3.19
^4^CH_2_	33.9	1.41; 1.94	^6a^C, ^8a^C	76.08; 76.12	–
^3^CH_2_	34.4	1.11; 1.87	^11a^C	133.5	
^6^CH_2_	36.4	1.45; 1.63	^11b^C	137.5	
^9^CH_2_	40.9	1.96; 2.01	CO_2_	172.7; 175.3	

**Figure 2:**
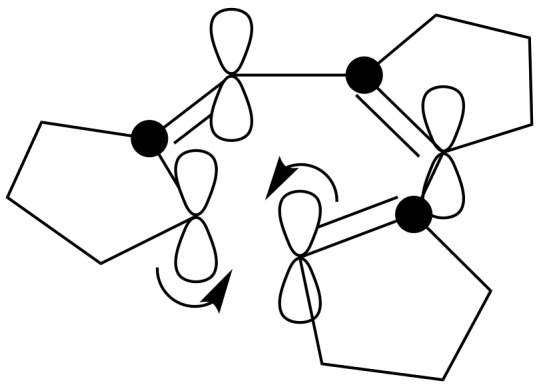
A possible mechanism for the *trans*-position of the methyne hydrogens in the azepine ring: the electrocyclization proceeds via a conrotatory mechanism due to the antisymmetry of the HOMO.

It is well-known that cyclopentanone is prone to self-condensation. In the presence of ammonia these reactions may lead to heterocycle formation [11]. Presumably, a prototropic shift in the enamine-imine intermediate **4** is followed by electrocyclization to the cyclic azomethine ylide, which then reacts with dimethyl fumarate in a 1,3-dipolar cycloaddition. The suggested mechanism accounts for the *trans*-position of the methyne hydrogens in the azepine ring: electrocyclization proceeds via a conrotatory mechanism due to the antisymmetry of the HOMO (Figure 2).

**Figure 3:**
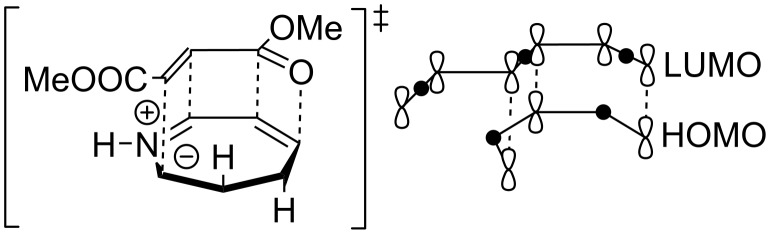
Selective formation of a single diastereomer in the 1,3-dipolar cycloaddition reaction.

The selective formation of a single diastereomer in the 1,3-dipolar cycloaddition reaction is likely a result from secondary interactions of orbitals of the π-systems of the dipole and dipolarophile (Figure 3).

#### Nitroxide

**Scheme 3:**
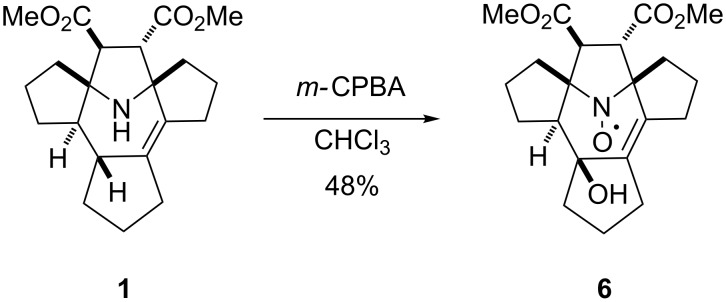
Synthesis of nitroxide **6**.

**Figure 4:**
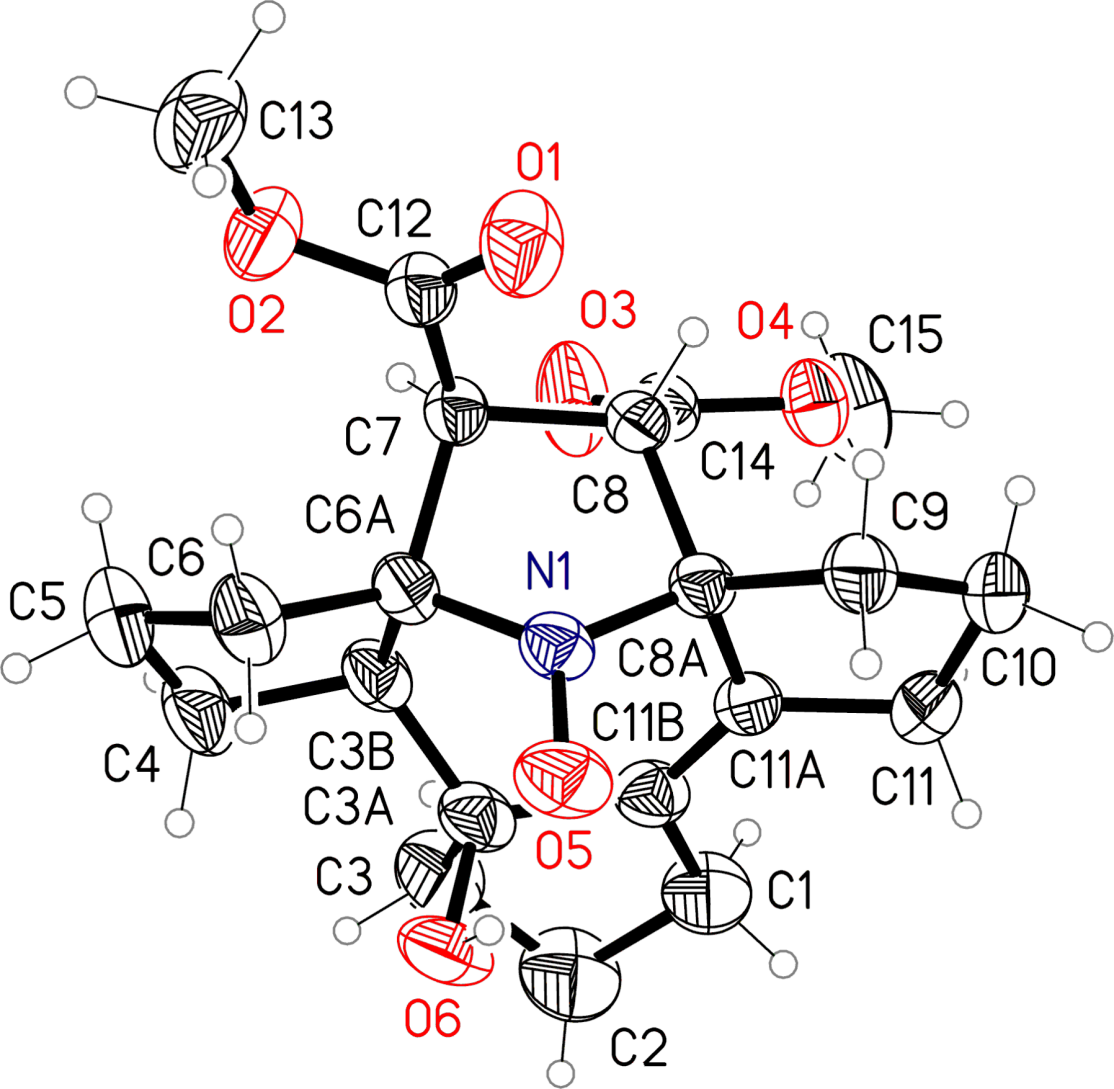
X-ray structure of compound **6** (one of the two enantiomers present in the crystal).

**Scheme 4:**
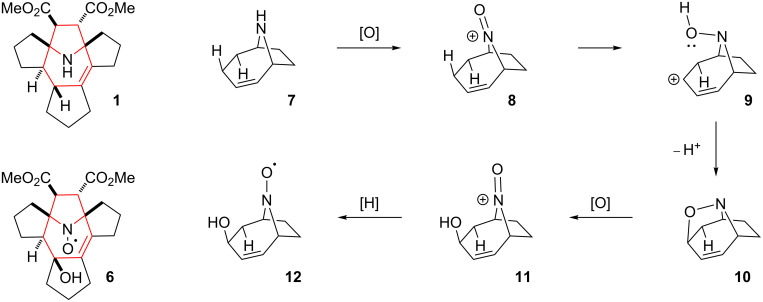
A proposed mechanism for nitroxide **6** synthesis.

Oxidation of **1** with *m*-CPBA afforded the nitroxide **6** with 48% yield (Scheme 3). It is noteworthy that the oxidation of the amino group is accompanied by the stereospecific hydroxylation at position 4 of the 2,3,4,7-tetrahydroazepine ring. The structure assignment was based on the single-crystal X-ray analysis (Figure 4) and a possible mechanism for this hydroxylation is shown in Scheme 4. Oxidation of amines with peracids is known to proceed through oxoammonium cation formation [12]. The close proximity of this reactive group to the allyl hydrogen results in hydride abstraction with the formation of carbocation **9** and subsequent cyclization to the bicyclic alkoxyamine **10**. The resulting isoxazolidine ring is then opened with *m*-CPBA in the usual way, retaining the configuration of the asymmetric center at ^3a^C-OH [13] (Scheme 4).

The steric strain in molecules **1** and **6** is characterized by an elongation of the bonds C6A–C7 1.579(2), C8–C8A 1.573(2) Å in **1** and C3B–C6A 1.580(2), C8–C8A 1.573(2) Å in **6**. The conformation of the azepane ring in **1** is close to a distorted boat with a kink at the C3A–N1 line, while in **6** the conformation of this ring is close to a distorted half-chair. Presumably, this difference is due to the formation of an intramolecular hydrogen bond O6–H^…^O5 (H^…^O 1.95(2) Å, O–H^…^O 154(2)°) in molecule **6**. In the crystal of **1** the amino group participates in intermolecular hydrogen bonding N1–H^…^O3 (H^…^O 2.39(2) Å, N–H^…^O 167(1)°) forming chains of molecules along the *a-*axis.

#### EPR measurements

**Figure 5:**
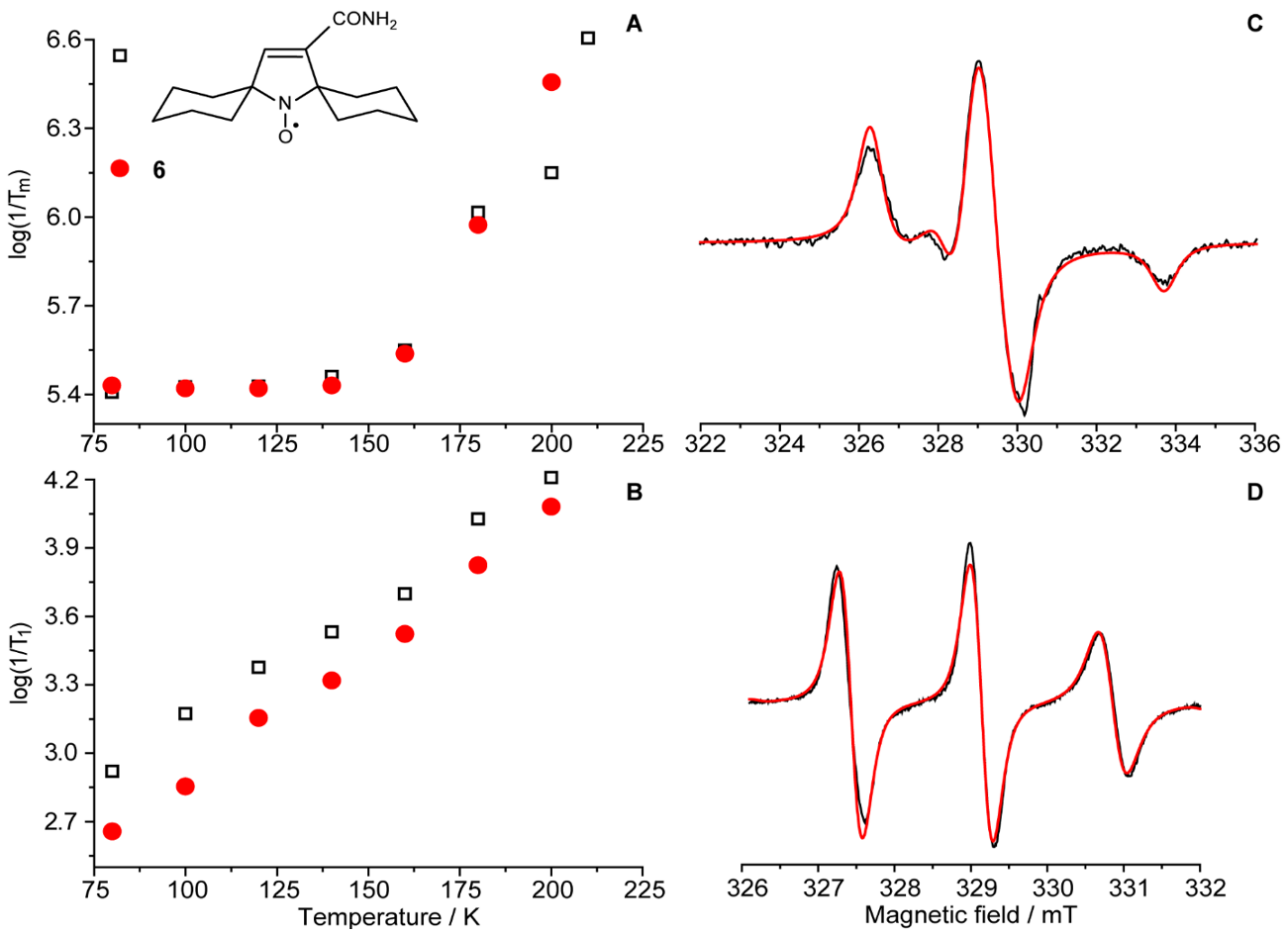
A, B) Temperature dependence of the electron spin relaxation times in water/glycerol at X-band frequency. The solid red dots are for nitroxide **6**; the open black squares are for spirocyclohexane-substituted nitroxide. C, D) CW EPR spectra of nitroxide **6** at 180 K (C) and at room temperature (D) in water/glycerol. Black lines are experimental spectra and the red lines are simulations for hfi, g-factor and correlation times of A(N) = [0.71; 0.71; 3.71] mT, g = [2.0087; 2.0055; 2.0019], *t*_corr_ = 0.53 ns (at 298 K).

Figure 4 demonstrates the X-band CW EPR spectra of nitroxide **6** in water/glycerol solution at 180 K and at room temperature with simulations (red) using the parameters listed in the caption. The electron spin relaxation of nitroxides with different bulky substituents has been studied in water/glycerol solutions at low temperatures in numerous papers [9,14–16]. Previously, we have investigated the electron spin relaxation of a series of nitroxides with different bulky substituents in water solution and in trehalose [10]. Because **6** is a new class of a hindered nitroxide, we investigated its electron spin relaxation properties in water/glycerol solution which is the solvent of choice for biomolecular distance measurements by PELDOR/DEER. If the rotation of the radical is prevented, the primary relaxation mechanisms are (i) modulation of the ^14^N hyperfine interaction (hfi) anisotropy of the NO group by librational motion, and (ii) modulation of the hfi with other nuclei in the radical by rotation of the groups containing those nuclei (e.g., rotation of methyl groups). The temperature dependence of spin relaxation reveals the relevant mechanism. For comparison, relaxation of a spirocyclohexane-substituted nitroxide [10] is also shown in Figure 5. The *T*_m_ vs *T* dependence generally shows consistent trends in frozen solutions. For 2,5-tetramethyl-substituted pyrrolidine and piperidine nitroxides, a local maximum appears in their phase relaxation (1/*T*_m_) at *T* > 100 K as thermally activated rotation of their methyl groups becomes rapid. Above 140 K this rotation is rapid enough to average the hfi anisotropy and to cause some decrease in the phase relaxation rates. Finally, at *T* > 220 K the librational motion of the NO group dominates phase relaxation and causes the relaxation rate to increase with temperature in soft matter [14,17]. Consequently, the common tetramethyl-substituted nitroxides are characterized by a local bell-shaped maximum in their phase relaxation at *T* > 100 K, that is absent in more hindered nitroxides. In contrast, no bell-like shape is observed in the phase relaxation of nitroxides with two spirocyclohexane moieties adjacent to the N-O^•^ group [8–10] or for nitroxide **6**. The temperature dependence of 1/*T*_m_ is similar for both radicals in Figure 5 with some divergence above 180 K.

## Conclusion

An unexpected formation of a highly strained polycyclic amine from cyclopentanone, dimethyl fumarate and ammonium acetate was observed, and this amine was converted to a sterically-shielded polycyclic nitroxide. The temperature dependence of the electron spin relaxation of the nitroxide in water/glycerol solution is very similar to that of the previously studied nitroxides with two spirocyclohexane moieties adjacent to the N-O• group despite their very different structures.

## Experimental

^1^H NMR spectra were recorded at 400 or 600 MHz, and ^13^C NMR spectra were recorded at 100 or 150 MHz, respectively. ^1^H and ^13^C NMR chemical shifts (δ) were internally referenced to the residual solvent peak. IR spectra were acquired on an FTIR spectrometer in KBr and are reported in wavenumbers (cm^−1^). Reactions were monitored by TLC using UV light 254 nm, 1% aqueous permanganate and Dragendorff reagent as visualizing agents. Column chromatography was performed on silica gel 60 (70–230 mesh). X-ray diffraction data were obtained with a Bruker KAPPA APEX II diffractometer using ϕ, ω scans with Mo Kα radiation (λ = 0.71073 Å) and a graphite monochromator. CCDC 1947797 (for **1**) and 1947798 (for **6**) contain the supplementary crystallographic data for this paper. These data can be obtained free of charge from The Cambridge Crystallographic Data Centre at http://www.ccdc.cam.ac.uk.

### Synthesis of dimethyl 2,3,3a,3b,4,5,6,7,8,9,10,11-dodecahydro-1*H*-6a,8a-epiminotricyclopenta[*a*,*c*,*e*][8]annulene-7,8-dicarboxylate (**1**)

A mixture of ammonium acetate (616 mg, 8 mmol), cyclopentanone (1.3 mL, 15 mmol), dimethyl fumarate (576 mg, 4 mmol) and benzene (10 mL) was placed in a Dean–Stark apparatus and stirred under reflux for 48 h. The solvent was distilled off in vacuum and the residue was dissolved in ethyl acetate. The organic layer was washed with 5% sodium hydrogen carbonate solution and then extracted with 5% sulfuric acid. Acidic extracts were basified with Na_2_CO_3_ and extracted with ethyl acetate. The extract was dried with Na_2_CO_3_ and the solvent was distilled off in vacuum to give a dark oil, which was purified using column chromatography on silica gel (hexane/ethyl acetate 4:1). **1**: 70 mg (5%); colorless crystals; mp 146.8–148.4 °C; IR (KBr): 3298 (N-H), 1734, 1718 (C=O); ^1^H NMR (400 MHz, CDCl_3_, δ) 1.08–1.16 (m, 1H), 1.35–1.48 (m, 3H), 1.49–1.57 (m, 1H), 1.60–1.69 (m, 3H), 1.70–1.76 (m, 1H), 1.84–1.90 (m,1H), 1.91–1.98 (m, 2H), 1.99–2.08 (m, 3H), 2.14–2.20 (m, 1H), 2.20–2.29 (m, 1H), 2.36–2.41 (m, 1H), 2.42–2.49 (m, 1H), 2.58–2.80 (br, 1H), 3.19 (d *J*_d_ = 3.1 Hz, 1H), 3.44 (d *J*_d_ = 3.1 Hz, 1H), 3.57 (s, 3H), 3.66 (s, 3H); ^13^C{^1^H} NMR (100 MHz, CDCl_3_, δ) 21.0, 24.0, 25.3, 30.0, 32.3, 33.8, 34.3, 36.3, 40.8, 45.9, 51.4, 51.5, 55.0, 59.8, 60.4, 76.0, 76.1, 133.5, 137.4, 172.7, 175.3; Anal. calcd for С_21_H_29_NO_4_: C, 70.17; H, 8.13; N, 3.90; found: C, 69.84; H, 7.89; N, 3.96. X-ray: triclinic system, *P*-1, *a* = 6.2467(4), *b* = 9.2039(8), *c* = 16.7575(15) Å, α = 98.858(4), β = 94.176(3), γ = 95.362(3)º, *V* = 944.07(13) Å^3^, *Z* = 2, 2θ_max_ = 27.271°, 4168 independent reflections (*R*_int_ 0.0429), *R*_1_ 0.0602, *wR*_2_ 0.1696, S 1.030 [for 3234 *I* > 2σ(*I*)].

### Synthesis of 3a-hydroxy-7,8-bis(methoxycarbonyl)-2,3,3a,3b,4,5,6,7,8,9,10,11-dodecahydro-1*H*-6a,8a-epiminotricyclopenta[a,c,e][8]annulene *N*-oxyl (**6**)

A 70% *m*-CPBA solution (200 mg, 0.9 mmol) was added to a solution of amine **1** (108 mg, 0.3 mmol) in chloroform (2 mL) and stirred at room temperature for 30 min. The reaction mixture was washed 3 times with a 5% solution of sodium bicarbonate. The extract was dried with Na_2_CO_3_ and the solvent was distilled off in vacuum to give a red oil, which was purified using column chromatography on silica gel (hexane/ethyl acetate 4:1). **2**: 56 mg (48%); red crystals; mp 127.0 °C dec, IR (KBr): 3438 (O-H) 1730 (C=O); X-ray: triclinic system, *P*-1, *a* = 8.2240(5), *b* = 10.9423(6), *c* = 11.4515(8) Å, α = 83.440(3), β = 75.611(3), γ = 83.119(2)º, *V* = 987.17(11) Å^3^, *Z* = 2, 2θ_max_ = 27.292°, 4388 independent reflections (*R*_int_ 0.0375), *R*_1_ 0.0439, *wR*_2_ 0.1215, S 1.011 [for 3451 *I* > 2σ(*I*)].

### EPR measurements

Continuous wave (CW) EPR measurements were carried out using a commercial Bruker Elexsys E540 X-band spectrometer. The CW EPR spectra were simulated using EasySpin [18]. Pulse EPR experiments were carried out using a commercial Bruker Elexsys E580 X/Q-band spectrometer equipped with an Oxford helium flow cryostat and temperature control system. An ER 4118X-MD5W resonator was used for X-band measurements. *T*_m_ was measured using a two-pulse electron spin echo (ESE) sequence; *T*_1_ was measured using the inversion-recovery technique with a π-pulse for inversion and a two-pulse ESE sequence for detection. The π-pulse lengths were nominally 20 ns.

## Supporting Information

File 1 Copies of the IR and ^1^H, ^13^C, HMBC, HSQC NMR spectra and X-ray analysis data.

## References

[1] Tietze L F (2014). Domino reactions: concepts for efficient organic synthesis.

[2] Hill M D (2010). Chem – Eur J.

[3] Estévez V, Villacampa M, Menéndez J C (2014). Chem Soc Rev.

[4] Dobrynin S A, Glazachev Y I, Gatilov Y V, Chernyak E I, Salnikov G E, Kirilyuk I A (2018). J Org Chem.

[5] Karthikeyan G, Bonucci A, Casano G, Gerbaud G, Abel S, Thomé V, Kodjabachian L, Magalon A, Guigliarelli B, Belle V (2018). Angew Chem, Int Ed.

[6] Bleicken S, Assafa T E, Zhang H, Elsner C, Ritsch I, Pink M, Rajca S, Jeschke G, Rajca A, Bordignon E (2019). ChemistryOpen.

[7] Meyer V, Swanson M A, Clouston L J, Boratyński P J, Stein R A, Mchaourab H S, Rajca A, Eaton S S, Eaton G R (2015). Biophys J.

[8] Rajca A, Kathirvelu V, Roy S K, Pink M, Rajca S, Sarkar S, Eaton S S, Eaton G R (2010). Chem – Eur J.

[9] Kathirvelu V, Smith C, Parks C, Mannan M A, Miura Y, Takeshita K, Eaton S S, Eaton G R (2009). Chem Commun.

[10] Kuzhelev A A, Strizhakov R K, Krumkacheva O A, Polienko Y F, Morozov D A, Shevelev G Y, Pyshnyi D V, Kirilyuk I A, Fedin M V, Bagryanskaya E G (2016). J Magn Reson.

[11] Edgar O B, Johnson D H (1958). J Chem Soc.

[12] Sen' V D, Golubev V A, Efremova N N (1982). Russ Chem Bull.

[13] Ali S A, Wazeer M I M (1993). Tetrahedron.

[14] Biller J R, Elajaili H, Meyer V, Rosen G M, Eaton S S, Eaton G R (2013). J Magn Reson.

[15] Kirilyuk I A, Polienko Y F, Krumkacheva O A, Strizhakov R K, Gatilov Y V, Grigor’ev I A, Bagryanskaya E G (2012). J Org Chem.

[16] Kirilina E P, Dzuba S A, Maryasov A G, Tsvetkov Y D (2001). Appl Magn Reson.

[17] Sato H, Kathirvelu V, Fielding A, Blinco J P, Micallef A S, Bottle S E, Eaton S S, Eaton G R (2007). Mol Phys.

[18] Stoll S, Schweiger A (2006). J Magn Reson.

