# Supplementary material for: Unexpected one-pot formation of the 1H-6a,8a-epiminotricyclopenta[a,c,e][8]annulene system from cyclopentanone, ammonia and dimethyl fumarate. Synthesis of highly strained polycyclic nitroxide and EPR study
Source: Beilstein J Org Chem. 2019 Nov 7;15:2664–70. doi: 10.3762/bjoc.15.259 (PMC6880831; doi:10.3762/bjoc.15.259)
Supplement: File 1 — Copies of the IR and 1H, 13C, HMBC, HSQC NMR spectra and X-ray analysis data. [file Beilstein_J_Org_Chem-15-2664-s001.pdf]

BEILSTEIN JOURNAL OF ORGANIC CHEMISTRY

Supporting Information

for

Unexpected one-pot formation of the 1*H*-6a,8a-epiminotricyclopenta[*a*,*c*,*e*][8]annulene system from cyclopentanone, ammonia and dimethyl fumarate. Synthesis of highly strained polycyclic nitroxide and EPR study

Sergey A. Dobrynin, Igor A. Kirilyuk, Yuri V. Gatilov, Andrey A. Kuzhelev, Olesya A. Krumkacheva, Matvey V. Fedin, Michael K. Bowman and Elena G. Bagryanskaya

Beilstein J. Org. Chem. **2019,** *15,* 2664–2670. doi:10.3762/bjoc.15.259

Copies of the IR and ^{1}H, ^{13}C, HMBC, HSQC NMR spectra and X-ray analysis data

License and Terms: This is a supporting information file under the terms of the Creative Commons Attribution License (http://creativecommons.org/licenses/by/4.0). Please note that the reuse, redistribution and reproduction in particular requires that the authors and source are credited. The license is subject to the *Beilstein Journal of Organic Chemistry* terms and conditions: (https://www.beilstein-journals.org/bjoc)

Table of contents

X-ray analysis data S2

Table S1 Crystallographic data and structure refinement parameters of dimethyl 2,3,3a,3b,4,5,6,7,8,9,10,11-dodecahydro-1*H*-6a,8a-epiminotricyclopenta[*a*,*c*,*e*][8]annulene-7,8-dicarboxylate (**1**) and 3a-hydroxy-7,8-bis(methoxycarbonyl)-2,3,3a,3b,4,5,6,7,8,9,10,11-dodecahydro-1*H*-6a,8a-epiminotricyclopenta[*a*,*c*,*e*][8]annulene *N*-oxyl (**6**). S2

^{1}H NMR spectra S3

^{1}H NMR (400 MHz, $CDCl_3$) of **1** S3

^{13}C{^{1}H} NMR (100 MHz, $CDCl_3$) of **1** S3

HMBC (600 MHz, $CDCl_3$) **1** S4

HSQC (600 MHz, $CDCl_3$) of **1** S4

COSY (600 MHz, $CDCl_3$) of **1** S5

IR spectra S6

IR (KBr) of **1** S6

IR (KBr) of **6**. S6

X-ray analysis data

X-ray diffraction experiments for the compounds **1** and **6** were performed on a Bruker KAPPA APEX II diffractometer (MoKα radiation, CCD detector, ω-φ scanning, 296 K). The absorption corrections were introduced with the SADABS program [1]. The structures were solved by direct methods and refined anisotropically (except H atoms) using the SHELX2014 programs [2]. The hydrogen atom positions were calculated geometrically and refined in riding model. H atoms at N1 (**1**) and O6 (**6**) were located from difference maps and refined isotropically.

1. Sheldrick G. M., SADABS V. 2.01, Bruker AXS Inc. *Madison, Wisconsin, USA*. **2004**.

2. Sheldrick G. M. SHELXT–Integrated space-group and crystal-structure determination. *Acta Crystallogr., Sect. A: Found. Adv.* **2015**, 71, 3-8.

Table S1: Crystallographic data and structure refinement parameters of dimethyl 2,3,3a,3b,4,5,6,7,8,9,10,11-dodecahydro-1*H*-6a,8a-epiminotricyclopenta[*a*,*c*,*e*][8]annulene-7,8-dicarboxylate (**1**) and 3a-hydroxy-7,8-bis(methoxycarbonyl)-2,3,3a,3b,4,5,6,7,8,9,10,11-dodecahydro-1*H*-6a,8a-epiminotricyclopenta[*a*,*c*,*e*][8]annulene *N*-oxyl (**6**).

Parameter	**1**	**6**
Chemical formula	$C_{21}H_{29}NO_4$	$C_{21}H_{28}NO_6$
Formula weight	359.45	390.44
Temperature (K)	296	296
Crystal system	Triclinic	Triclinic
Space group	P-1	P-1
a (Å)	6.2467(4)	8.2240(5)
b (Å)	9.2039(8)	10.9423(6)
c (Å)	16.7575(15)	11.4515(8)
α (deg)	98.858(4)	83.440(3)
β (deg)	94.176(3)	75.611(3)
γ (deg)	95.362(3)	83.119(2)
Cell volume ($Å^3$)	944.07(13)	987.17(11)
Z	2	2
d_{calc} (g cm^{-3})	1.264	1.314
μ (mm^{-1})	0.087	0.096
Crystal dimensions (mm)	0.14×0.27×0.57	0.31×0.47×0.59
θ max (deg)	27.27	27.29
Reflections collected/ unique	20324/4168	25779
R (int)	0.0429	0.0375
Reflections with $I > 2\sigma(I)$	3234	3451
Parameters	241	258
Final R_1 ($I > 2\sigma(I)$)	0.0602	0.0439
Final wR_2 ($I > 2\sigma(I)$)	0.1696	0.1215
Final R_1 (all data)	0.0784	0.0585
Final wR_2 (all data)	0.2013	0.1390
Goodness-of-fit on F^2	1.030	1.011
CCDC number	1947797	1947798

1. NMR spectra

^{1}H NMR (400 MHz, $CDCl_3$) of dimethyl 2,3,3a,3b,4,5,6,7,8,9,10,11-dodecahydro-1*H*-6a,8a-epiminotricyclopenta[*a*,*c*,*e*][8]annulene-7,8-dicarboxylate (**1**)

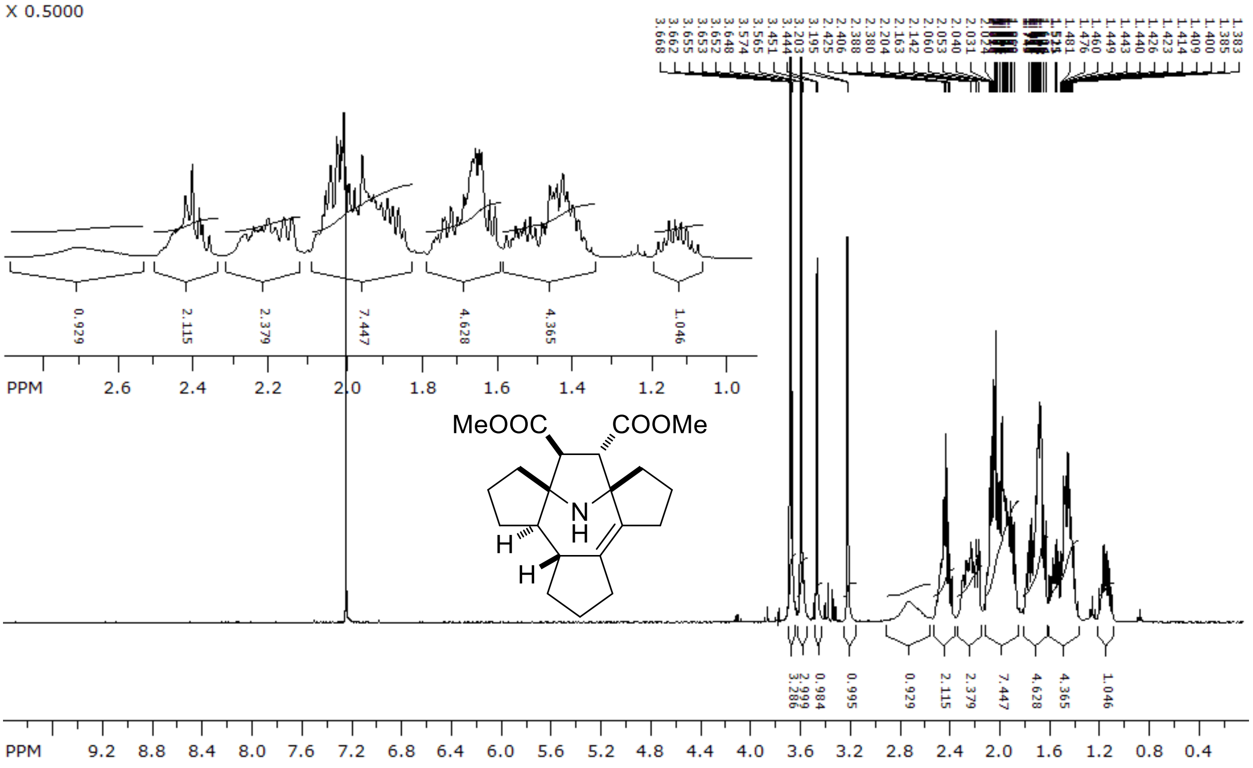

$^{13}C\{^{1}H\}$ NMR (100 MHz, $CDCl_3$) of dimethyl 2,3,3a,3b,4,5,6,7,8,9,10,11-dodecahydro-1*H*-6a,8a-epiminotricyclopenta[*a*,*c*,*e*][8]annulene-7,8-dicarboxylate (**1**)

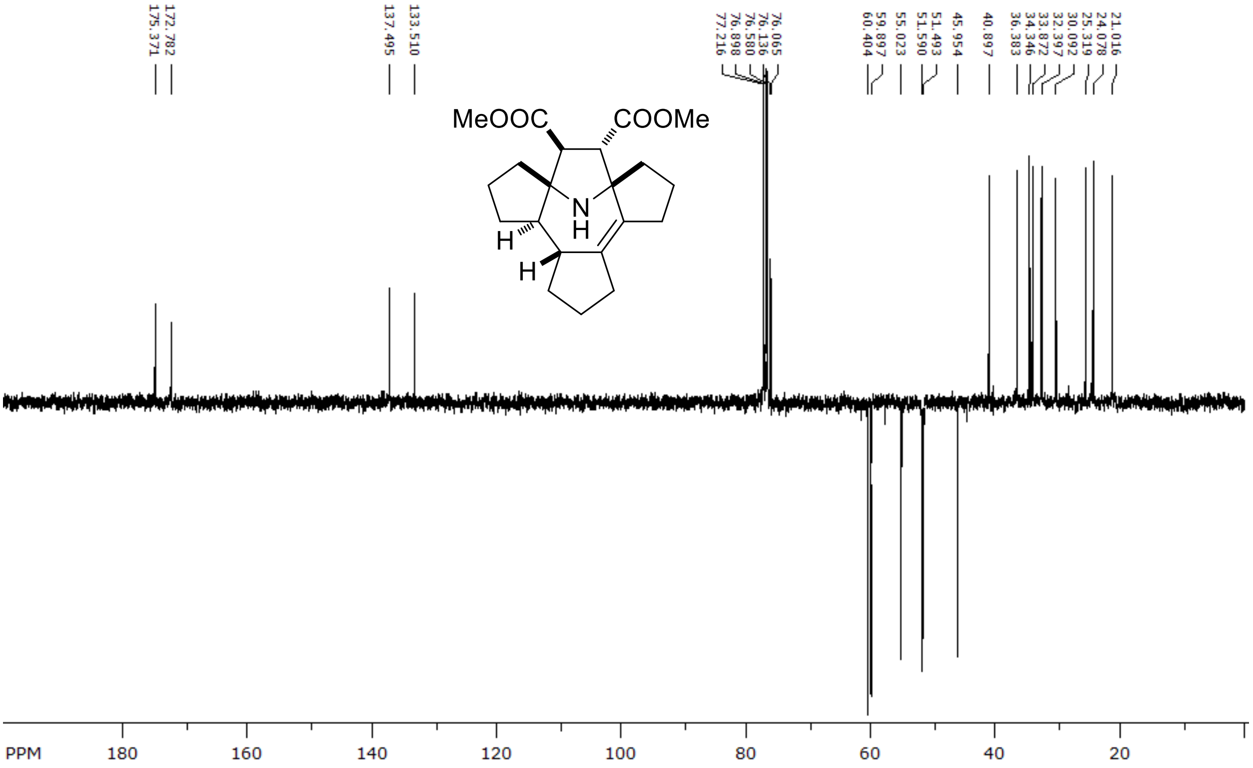

HMBC (600 MHz, $CDCl_3$) of dimethyl 2,3,3a,3b,4,5,6,7,8,9,10,11-dodecahydro-1*H*-6a,8a-epiminotricyclopenta[*a*,*c*,*e*][8]annulene-7,8-dicarboxylate (**1**)

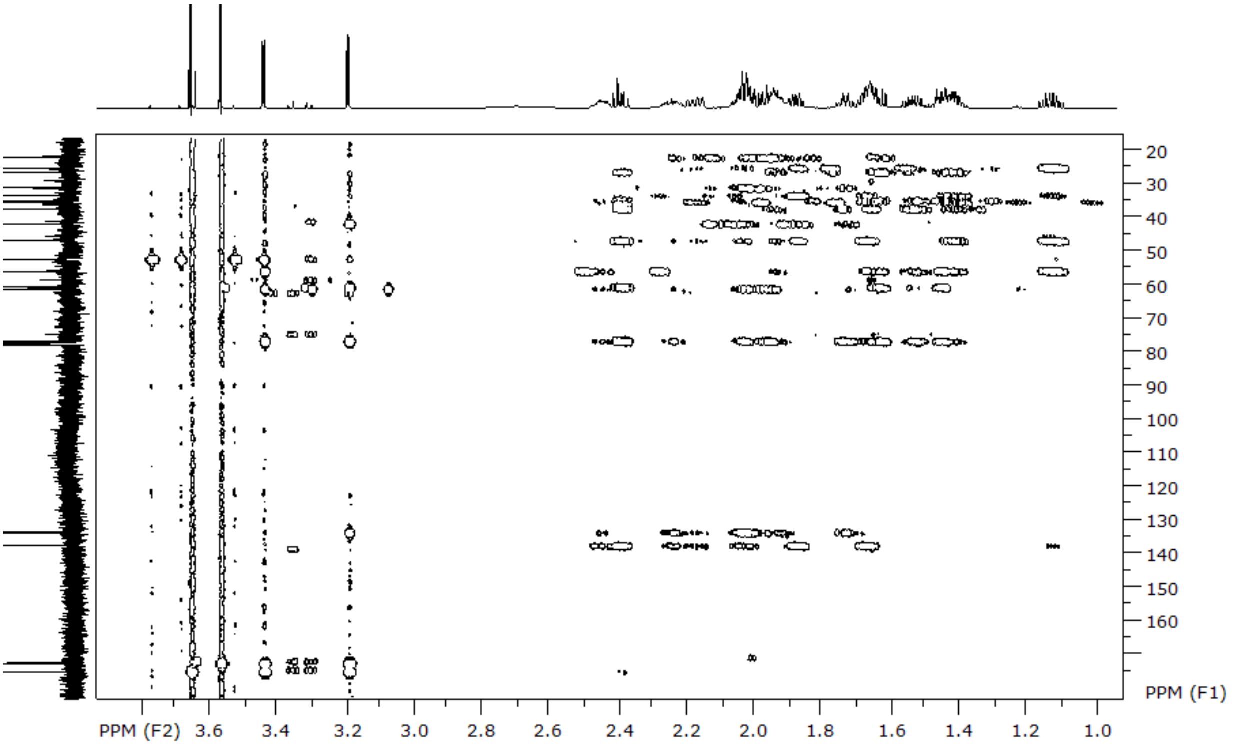

HSQC (600 MHz, $CDCl_3$) of dimethyl 2,3,3a,3b,4,5,6,7,8,9,10,11-dodecahydro-1*H*-6a,8a-epiminotricyclopenta[*a*,*c*,*e*][8]annulene-7,8-dicarboxylate (**1**)

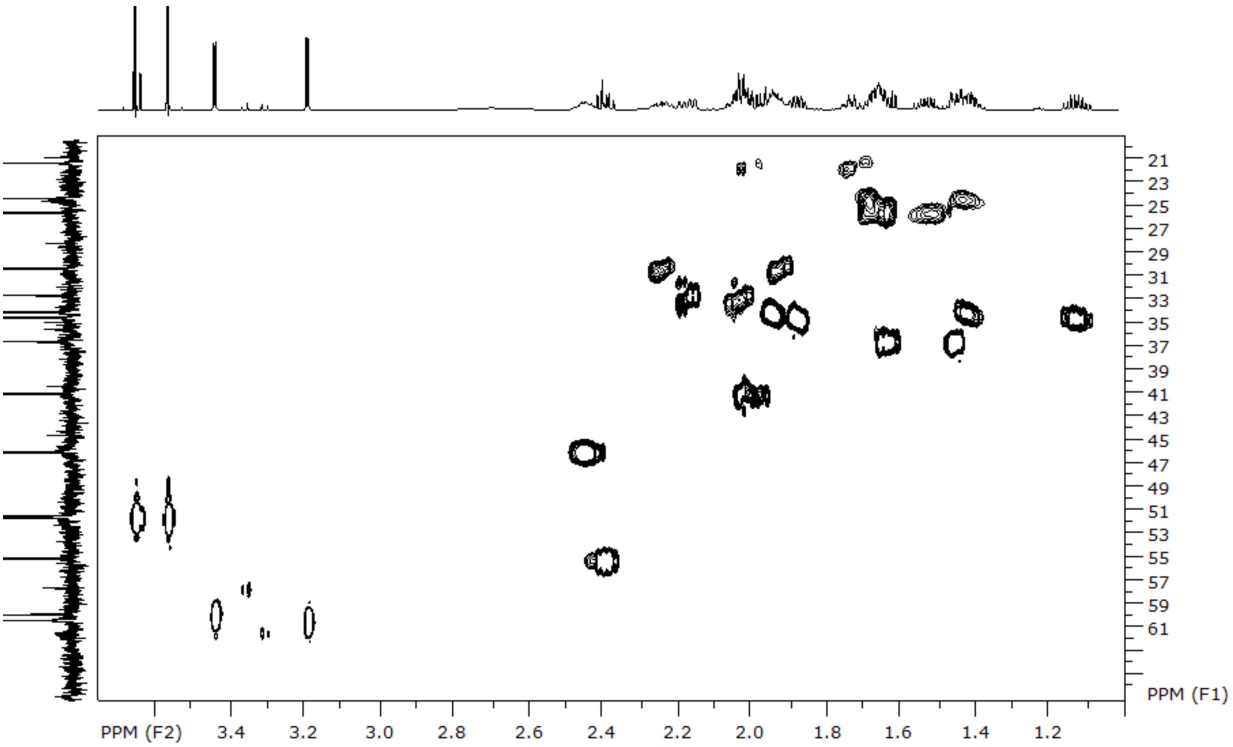

COSY (600 MHz, $CDCl_3$) of dimethyl 2,3,3a,3b,4,5,6,7,8,9,10,11-dodecahydro-1*H*-6a,8a-epiminotricyclopenta[*a*,*c*,*e*][8]annulene-7,8-dicarboxylate (**1**)

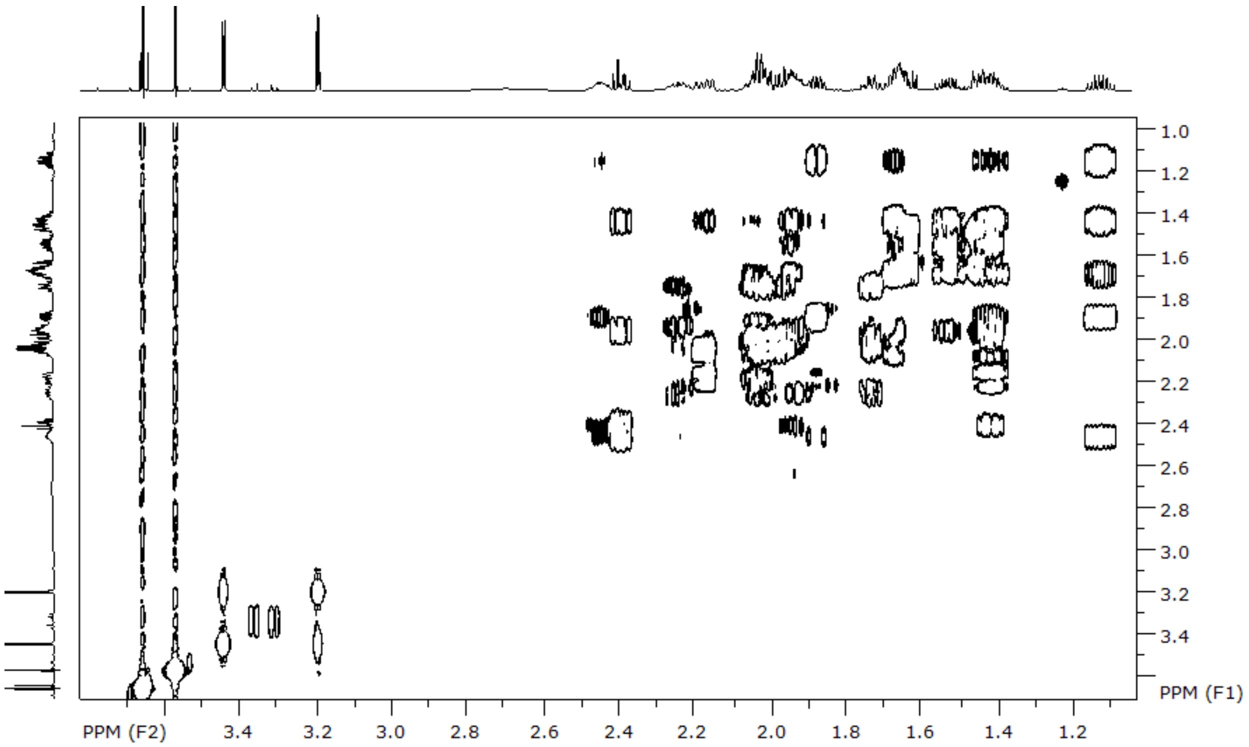

2. IR spectra

IR (KBr) of dimethyl 2,3,3a,3b,4,5,6,7,8,9,10,11-dodecahydro-1*H*-6a,8a-epiminotricyclo-penta[*a*,*c*,*e*][8]annulene-7,8-dicarboxylate (**1**)

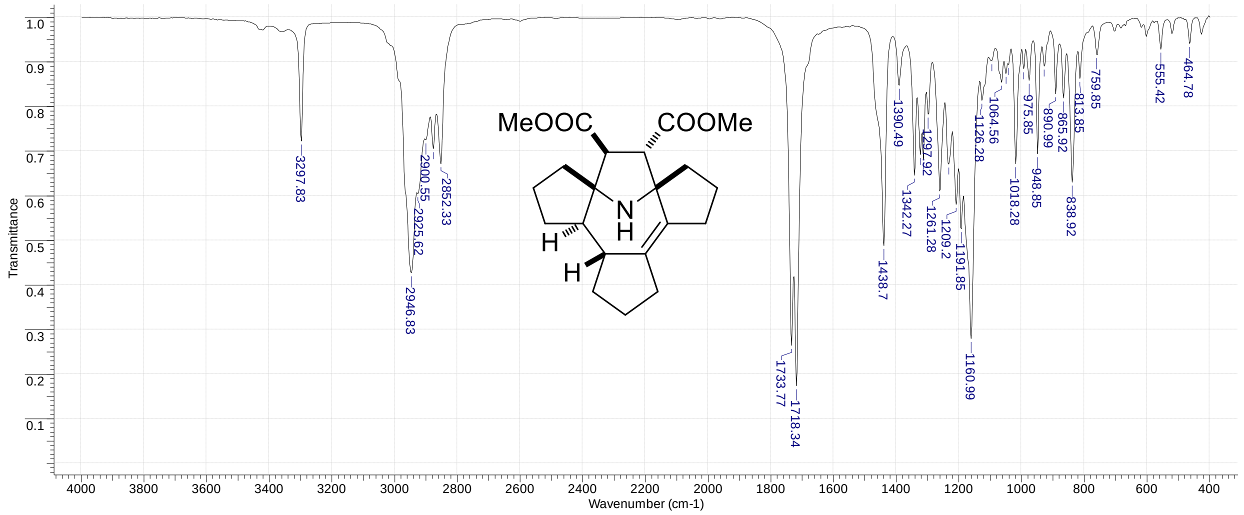

cm-1	cm-1	cm-1
3297.8	1313.3	1018.3
2946.8	1297.9	993.2
2925.6	1261.3	975.8
2900.5	1232.3	948.8
2877.4	1209.2	927.6
2852.3	1191.8	891.0
1733.8	1161.0	865.9
1718.3	1126.3	838.9
1438.7	1095.4	813.9
1390.5	1064.6	759.9
1342.3	1049.1	555.4
1323.0	1041.4	464.8

IR (KBr) of 3a-hydroxy-7,8-bis(methoxycarbonyl)-2,3,3a,3b,4,5,6,7,8,9,10,11-dodecahydro-1*H*-6a,8a-epiminotricyclopenta[*a*,*c*,*e*][8]annulene *N*-oxyl (**6**)

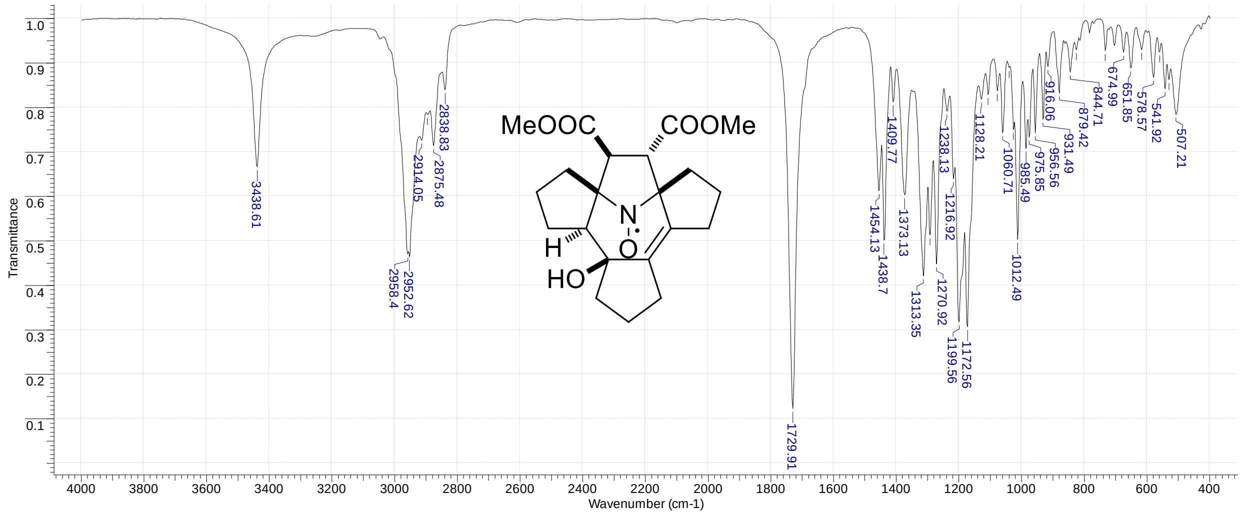

cm-1	cm-1	cm-1	cm-1
3438.6	1373.1	1060.7	825.4
2958.4	1313.3	1039.5	732.9
2952.6	1292.1	1026.0	675.0
2914.0	1270.9	1012.5	651.9
2894.8	1238.1	985.5	617.1
2875.5	1216.9	975.8	578.6
2838.8	1199.6	956.6	559.3
1729.9	1172.6	931.5	541.9
1454.1	1128.2	916.1	530.4
1438.7	1107.0	879.4	507.2
1409.8	1078.1	844.7	
